# Supplementary material for: Antifungal Potential of Azotobacter salinestris Strain Azt 31 against Phytopathogenic Fusarium spp. Associated with Cereals
Source: J Fungi (Basel). 2022 Apr 30;8(5):473. doi: 10.3390/jof8050473 (PMC9145299; doi:10.3390/jof8050473)
Supplement: Supplementary file 1 [file jof-08-00473-s001.zip › jof-1683962-supplementary.pdf]

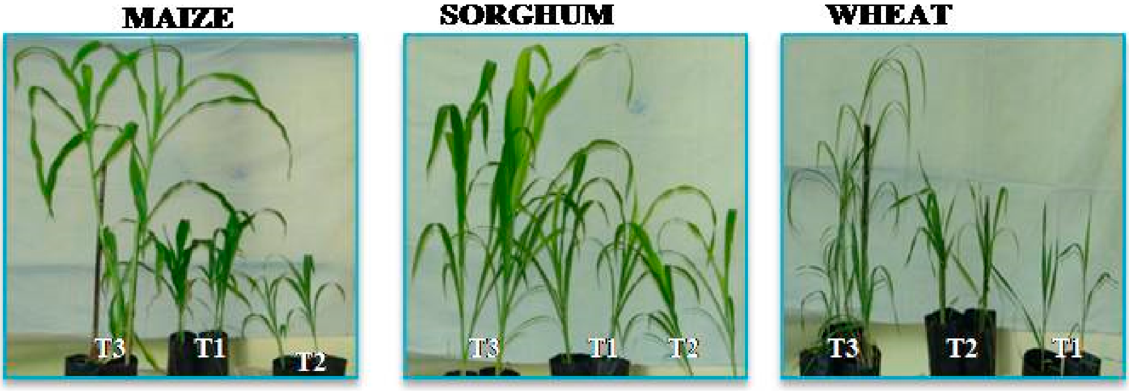

Supplementary Figure S1: Pot experiment showing the effect of *A. salinestris* on shoot length of cereals. A) Maize, B) Sorghum, and C) Wheat.

T1 is control; T2 is *F. sporotrichioides* alone; T3 is *A. salinestris* + *F. sporotrichioides*
